# Flow of Red Blood Cells in Stenosed Microvessels

**DOI:** 10.1038/srep28194

**Published:** 2016-06-20

**Authors:** Koohyar Vahidkhah, Peter Balogh, Prosenjit Bagchi

**Affiliations:** 1 Mechanical and Aerospace Engineering Department, Rutgers, The State University of New Jersey, Piscataway, NJ 08854, USA

## Abstract

A computational study is presented on the flow of deformable red blood cells in stenosed microvessels. It is observed that the Fahraeus-Lindqvist effect is significantly enhanced due to the presence of a stenosis. The apparent viscosity of blood is observed to increase by several folds when compared to non-stenosed vessels. An asymmetric distribution of the red blood cells, caused by geometric focusing in stenosed vessels, is observed to play a major role in the enhancement. The asymmetry in cell distribution also results in an asymmetry in average velocity and wall shear stress along the length of the stenosis. The discrete motion of the cells causes large time-dependent fluctuations in flow properties. The root-mean-square of flow rate fluctuations could be an order of magnitude higher than that in non-stenosed vessels. Several folds increase in Eulerian velocity fluctuation is also observed in the vicinity of the stenosis. Surprisingly, a transient flow reversal is observed upstream a stenosis but not downstream. The asymmetry and fluctuations in flow quantities and the flow reversal would not occur in absence of the cells. It is concluded that the flow physics and its physiological consequences are significantly different in micro- versus macrovascular stenosis.

Vascular stenosis is a term that is commonly used to describe narrowing of blood vessels. When the stenosis occurs in a large artery, for example, aorta, coronary and carotid arteries, the disease is referred to as atherosclerosis, or large vessel disease^1^. Research over the past several decades has established the important role of fluid flow in large vessel disease^2^,^3^,^4^,^5^. In recent years, however, there is an apparent paradigm shift in our understanding of vascular stenosis. It has been established now that stenosis can and often occur in smaller arteries (arterioles) with internal diameters ranging up to a few hundred microns^6^,^7^. The condition, known as microvascular disease, could have severe consequences. For example, in coronary microvascular disease or CMVD, a blockage occurs in the microvessels supplying blood to heart muscles^8^,^9^. In lacunar infarct, a blockage occurs in the smaller arteries supplying blood to the interior of the brain. Microvascular stenosis can also occur in retinal and renal microcirculation^7^,^10^,^11^. Flow blockage in small vessels can occur also by adherent leukocytes, gas emboli, etc^12^,^13^,^14^,^15^. Apart from physiological examples, *in vitro* microstenosis geometry are utilized in biomedical devices for various purposes, e.g., for cell separation^16^,^17^.

Fluid mechanics of a microvascular stenosis is expected to be very different from that of a macrovascular stenosis. It is because blood behaves as a Newtonian fluid in large arteries but as a non-Newtonian fluid in microvessels^18^. Continuum assumption breaks down as the vessel diameter approaches the cell size. Although attempts have been made to apply the continuum models to microvascular stenosis^19^,^20^, no study exists that considered multifile flow of deformable cells through the stenosis geometry. Illustrations of the cellular nature of blood flow in small vessels are the well known Fahraeus-Lindqvist and Fahraeus effects. The former refers to a reduction of the apparent blood viscosity with decreasing vessel diameter. The latter refers to a reduction of the red blood cell volume fraction or hematocrit, with decreasing vessel diameter^18^,^22^,^23^,^24^. The influence of a stenosis on the Fahraeus-Lindqvist and Fahraeus effects is unknown, and is one objective of this study.

Also unknown is the local hemodynamic condition at the stenosis that dictates the growth and progression of the plaque^1^,^2^,^3^,^4^,^5^,^25^. Endothelial cells (ECs) lining the inner wall of blood vessels are known to respond to local hemodynamic conditions, such as the wall shear stress and flow oscillation. The ECs mediate the growth of stenosis by suppressing the nitric oxide release^26^,^27^. EC response, along with complex flow conditions near a stenosis trigger thrombus formation via platelet aggregation and adhesion^28^,^29^,^30^. In large blood vessels the flow oscillations and shear gradients arise primarily from the interaction between flow pulsatility and stenosis geometry^1^,^2^,^3^,^4^,^5^. In contrast, in small vessels, the motion of individual blood cell causes temporal and spatial variations leaving their ‘footprints’ on ECs^31^. Such temporal and spatial flow oscillations in stenosed microvessels have not been quantified in the literature. Furthermore, experimental data on the thickness of the cell-free layer (CFL) exists for non-stenosed vessels^21^, but not for stenosed vessels. The thickness of the CFL is expected to greatly vary over the length of a stenosis, unlike a constant CFL thickness observed in non-stenosed vessels. Studies using microfluidic conduits with severe constrictions showed greatly enhanced plasma and cell separation and increased downstream CFL thickness^16^.

To address the issues raised above, we consider a computational study of the flow of deformable red blood cells in stenosed microvessels. To make a reasonably fast computation over various parameters, we keep the geometry rather small. It is shown that the discrete nature of the cell motion significantly affects the flow physics in microvascular stenosis. This has an apparently different physiological consequence than in macrovascular stenosis. The Fahraeus and Fahraeus-Lindqvist effects are observed to be significantly enhanced by the interaction of the cells and stenosis geometry. Further, several non-intuitive phenomena, such as a transient upstream flow reversal and several folds increase in flow fluctuations, are also observed. The present study apparently is the first one to consider the rheological and hemodynamic implications of the cellular blood flow in microvascular stenosis.

## Method

**Figure 1:**
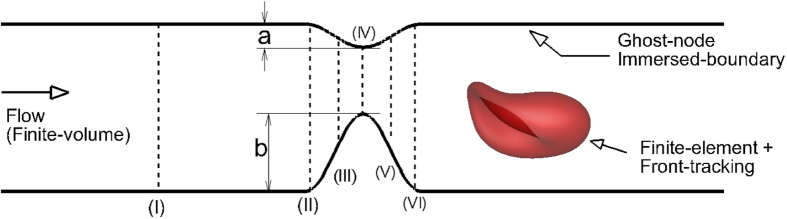
Geometry of a stenosed microvessel considered in the study. (I)–(VI) are streamwise locations where some flow quantities are measured for analysis.

The problem involves resolving the deforming boundaries of the red blood cells that are governed by complex mechanics. It also involves resolving the complex non-moving boundary defined by the vascular geometry. The numerical method has four modules: (i) a finite-element method for cell deformation, (ii) a finite-volume method for flow solver, (iii) a front-tracking method for coupling the fluid flow and the cell deformation, and (iv) a sharp-interface immersed-boundary method for coupling the stenosis geometry and the flow solver. The vessel geometry is shown in Fig. 1. We consider both axisymmetric and asymmetric constrictions. The axisymmetric constriction is defined by a cosine function of wavelength Ls and amplitude 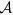 that is rotated about the centerline of the tube. For the asymmetric constriction, the amplitude of the cosine function is varied as the function is rotated. We consider a fixed *L*_*s*_*/D* = 0.7 where *D* is the nominal diameter away from the constriction. An area blockage 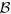, defined as the ratio of the blocked cross-sectional area at the throat of the constriction to the nominal cross-sectional area is varied up to 84%. The degree of (a)symmetry is denoted by 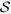 and is defined as *(b−a)/b* where *a* and *b* are the minimum and maximum values of 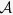 (see Fig. 1). Hence, 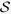 varies between 0 and 1, the lower limit representing a symmetric constriction.

Detailed description of the numerical method dealing with red blood cell deformation is given in our prior works^32^,^33^,^34^. Here a brief description is given for the sake of completeness. RBCs are modeled as capsules, i.e., viscous drops enclosed by zero-thickness elastic membranes. In this model, the ultrafine structure of the membrane, namely, the lipid bilayers and the spectrin network are indistinguishable. The resting shape is taken as the experimentally observed biconcave discocyte of end-to-end distance of 7.8 μm, surface area 134.1 μm^2^ and volume 94.1 μm^3^^35^. The fluids interior and exterior of the cells are assumed to be incompressible and Newtonian. The RBC membrane is assumed to resist shear deformation, area dilatation, and bending. The resistance against shear deformation and area dilatation is modeled following Skalak *et al*.^36^ and using an in-plane strain energy function *W*_*s*_ expressed as


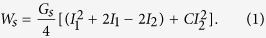


Here 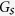 is the membrane shear elastic modulus, 
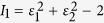
 and 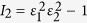 are the strain invariants of the Green strain tensor, and *ε*_1_ and *ε*_2_ are the principal stretch ratios. The Green strain tension is defined as 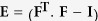 where 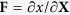 is the deformation gradient of the current configuration *x* relative to the original configuration **X** of the membrane. The parameter *C* controls the amount of area dilation and is chosen so that the total surface area change remains less than 1%. The principal elastic stresses (or tensions) are expressed as


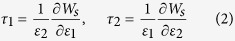


The bending resistance of the membrane is modeled following Helfrich’s formulation as^37^


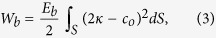


where *E*_*b*_ is the bending modulus, 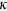 is the mean curvature, *c*_*o*_ is the spontaneous curvature, and 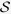 is the surface area.

A finite element method is used to obtain the membrane tension resulting from the shear deformation and area dilatation. The surface of each RBC is discretized using 20480 triangular elements (or, 10242 nodes). Each triangle is assumed to remain flat upon deformation. The displacement field v is assumed to vary linearly within each triangular element, and is expressed in terms of linear shape functions *N*_*i*_ as 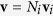 where the index *i* = 0, 1, 2 denotes the vertices of the triangle. The shape functions can be evaluated by knowing the coordinates of the vertices, and by letting, for example, *N*_1_ = 1 at the vertex 1 and 0 at vertices 2 and 3. Once the shape functions are known, the deformation gradient **F** is obtained. Subsequently, 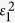 and 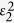 which are the eigenvalues of **F** . **F**^*T*^, and the stress tensor *τ* are obtained. Having the stress tensor in each element evaluated, we obtain the resultant elastic force at each node as


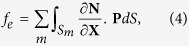


where **N** is the vector of the shape functions, 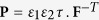 is the first Poila-Kirchhoff stress tensor, and *S*_*m*_ is the area of each of the *m* triangles surrounding the node.

An expression for a bending force density derived from Eq. 3 was used in numerical implementation:


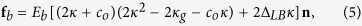


where 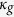 is the Gaussian curvature, Δ_*LB*_ is the Laplace-Beltrami operator, and **n** is the normal vector. The mean and Gaussian curvatures are evaluated at each vertex using a quadratic surface fitted to the vertex and its nearest neighboring vertices. Then, using the Gauss theorem 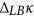 is approximated on a small surface patch *dS* as 
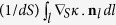
, where *l* denotes the patch boundary, 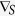 the surface gradient, and **n**_*l*_ the unit normal to the boundary *l*. The gradient 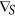 on a surface triangle can be obtained either by a linear interpolation of the surface and 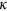, or using the Loop’s subdivision method.

The flow in the vessels is driven by a mean pressure gradient, and governed by the continuity and the Stokes equation as inertial effects are neglected. Once the membrane forces are computed, they are added to the Stokes equations as body forces using the delta functions that vanish away from the cell boundaries:


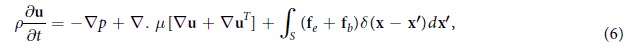


where *δ* is the three-dimensional Dirac-delta function, and **x** and **x**′ are the locations in the flow and on the cell surface, respectively. In numerical implementation, the delta function is approximated using a cosine function spanning over four grid points around the cell boundary. The fluid motion is solved utilizing a finite-volume discretization on a fixed (Eulerian) rectangular Cartesian staggered-grid. A projection-based time-split scheme is used wherein the momentum equation is solved in two steps as diffusion equation and pressure correction. The Eulerian resolutions used are 320 × 80 × 80, 480 × 120 × 120, and 640 × 160 × 160. Periodic conditions are enforced at the entrance and exit boundaries. Once the flow field is obtained at any time instance, the RBC membrane velocity **u**_*m*_ is obtained by interpolating the Eulerian velocity **u** using the delta function as noted above. The membrane is then advected as *d***x**′/*dt* = **u**_*m*_ to obtain its new shape and position.

Within the framework of the general solution methodology, the tube wall and stenosis are represented using a sharp-interface immersed boundary method^38^. The basic premise of this method is to use a non-body conforming mesh and enforce the no-slip boundary condition at the boundary by specifying an appropriate velocity at Eulerian grid points (ghost nodes, or GN) immediately outside the flow domain. The value to be imposed at each GN is determined using a boundary intercept (BI) and an image point (IP). The BI is the point on the immersed boundary that is closest to the GN, while the IP is the mirror image of the GN in the fluid domain, across the BI. For the velocity, the boundary condition to be imposed at the BI is of a Dirichlet type; it is taken to be the average of values at the GN and IP. In practice the interpolation stencil for a particular GN often includes the GN itself and/or other GN. Thus a linear system may need to be solved to obtain the GN velocity. For the projection operator, the boundary condition to be imposed is of a Neumann type. The presence of the immersed boundary modifies the discrete Laplacian of the projection operator and results in a non-symmetric linear system. A Flexible Generalized Minimum Residual (FGMRES) method with a multigrid V-cycle at the preconditioning step is employed for a fast and efficient solution of the linear system.

The length of the vascular segment simulated is *L/D* = 4.33. The relevant parameters are the vessel diameter *D*, the area blockage 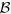, the degree of asymmetry 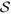, the tube hematocrit *H*_*t*_, and the driving mean pressure gradient *β*. The parameter β is defined as 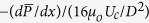 where *U*_*c*_ is an arbitrary velocity and *μ*_*o*_ is the plasma viscosity. In absence of the cells, *β* = 1 results in the Poiseuille flow with center-line velocity *U*_*P*_ equal to *U*_*c*_. All velocities are scaled by *U*_*c*_. Flow rates are scaled by [*U*_*P*_ (*D*/2π)^2^]. Simulations are done with dimensionless variables. The capillary number is defined as 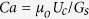. We keep *Ca* constant, but alter β to directly vary the mean flow in our simulations. The choice of β as the controlling parameter in our simulations is motivated by the fact that the mean pressure difference is used as the controlling parameter during *in-vitro* (glass tube) experiments (e.g.^39^). The membrane shear modulus *G*_*s*_ and bending stiffness *E*_*b*_ used in the simulations are 2.5 × 10^−6^ N/m and 6 × 10^−19^ J, respectively. We consider *D* = 11, 17 and 25 μm, 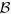 = 0, 50, 68 and 84%, 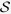 = 0, 0.3, 0.7 and 1, *H*_*t*_ ≈ 0, 11, 17 and 23%, and *β* = 1, 2, 3 and 4. The number of cells ranges from 5 to 132, depending on vessel size and hematocrit. For each *D*, *H*_*t*_ and *β*, cell motion in non-stenosed vessels is also simulated. About 90 total simulations are performed over the parameter space noted.

It should be noted that the pulsatility of blood flow arising from the beating of the heart is not significant in microcirculation as it is damped out as it travels down the vascular tree. Therefore, the base flow is assumed to be steady here.

## Results and Discussion

**Figure 2:**
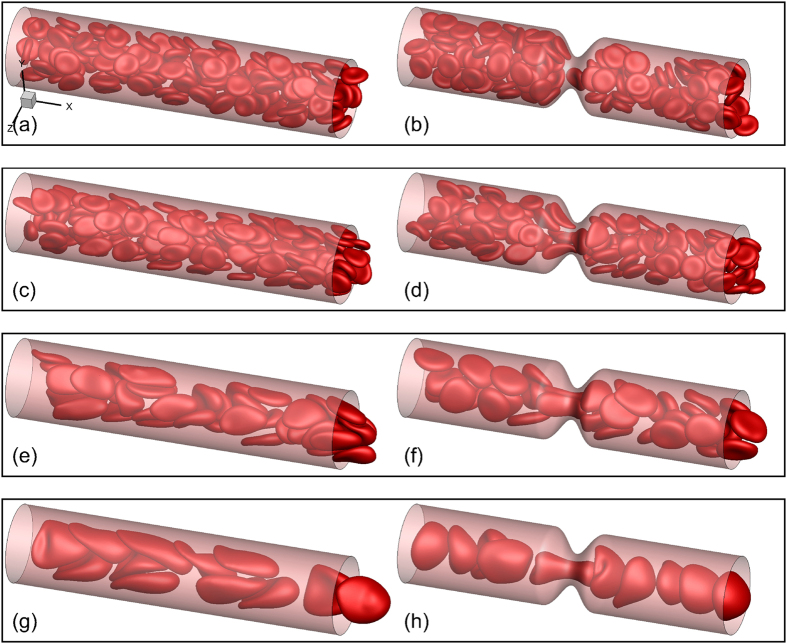
Snapshots showing instantaneous RBC distribution from a few representative simulations for non-stenosed (left column) and 84% stenosed (right column) vessels at *H*_*t*_ ≈ 22–24%. (**a**,**b**) *β* = 1, (**c–h**) *β* = 4.

Figure 2 shows snapshots for a few representative cases. Some general observations can be made here. Cells deform significantly in smaller vessels where they assume the slipper and parachute shapes as observed in experiments and simulations^40^,^41^,^42^,^43^,^44^. In larger vessels, the resting biconcave shape is somewhat maintained at low flow rates, but not at high flow rates. For the range of vessel size considered here, the RBCs flow in multifiles. The presence of a stenosis causes a geometric focusing of the cells. For the smallest tube considered (*D* = 11 μm), the multi-file motion that would occur without a stenosis is now converted to a single-file motion. Cells are significantly deformed as they squeeze before entering the stenosis, and bounce back upon exit. While most cells assume the slipper shape in the non-stenosed tube (*D* = 11 μm), nearly all assume the parachute shape in the stenosed tube. At a lower flow rate, crowding of the cells upstream the stenosis is also observed in larger vessels.

**Figure 3:**
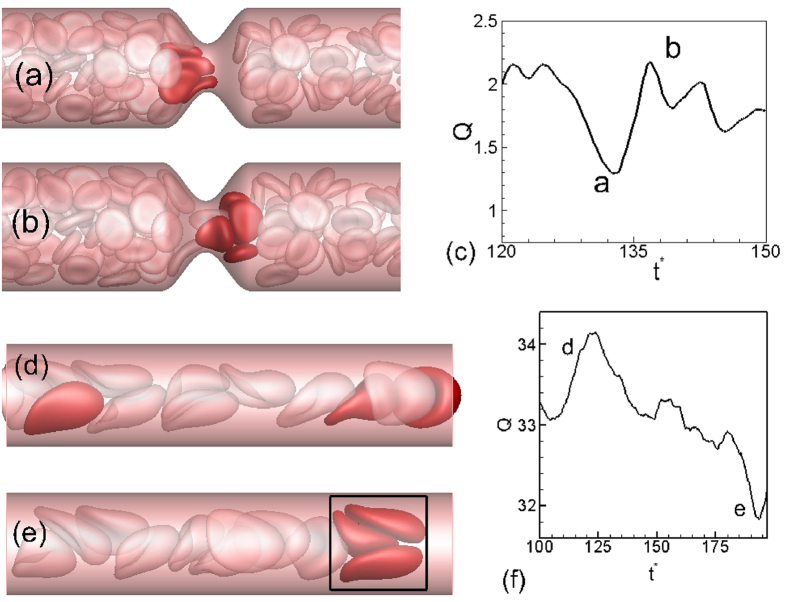
(**a,b**) Cell distribution in the vicinity of the stenosis at two instances corresponding to local extrema in instantaneous flow rate *Q*(*t*) shown in (**c**). (**d,e**,**f**) are for a non-stenosed vessel.

The discrete motion of cells results in large oscillations in flow rate over time. In Fig. 3, a close-up of the cell motion is shown at two time instants. The flow rate reaches a local minimum when multiple cells simultaneously attempt to enter the throat thereby blocking the flow area. A small region downstream the throat that is void of the RBCs is observed at this instant. As the cell cluster squeezes out of the throat, the flow rate increases. The flow oscillations become more regular (but still aperiodic) for the smallest diameter due to the single-file motion. Flow oscillations also exist in non-stenosed vessels, albeit of much reduced magnitude, and for a different reason. It is due to the formation of cell clusters as shown in Fig. 3(d–f). The fast moving cells near the center of the vessels push the slower moving cells further towards the wall reducing the thickness of the CFL and thereby reducing the flow rate momentarily. It is interesting to note that a steady flow is established in absence of the cells in both stenosed and non-stenosed vessels.

**Figure 4:**
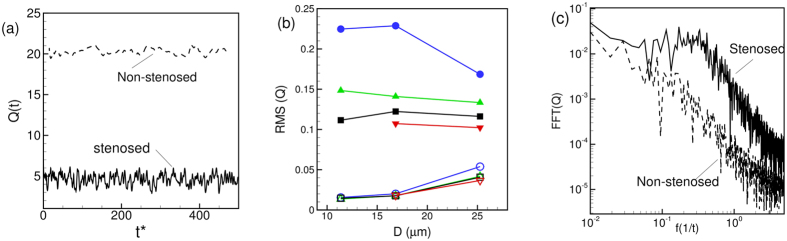
(**a**) Representative time-dependent flow rate *Q*(*t*) in stenosed (—) and non-stenosed (- - - - -) vessels; (**b**) RMS of fluctuations of 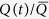 versus vessel diameter for *β* = 1 (O), 2 (Δ), 3 (◻), and 4 (∇); filled symbols are for stenosed vessels, and unfilled symbols for non-stenosed vessels. (**c**) Representative FFT of flow rate 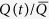 for stenosed (—) and non-stenosed (- - - - -) vessels.

Time-dependent flow rates *Q*(*t*) for representative simulations are shown in Fig. 4(a) over a long time. For the stenosed vessels, oscillations have a higher magnitude, and they occur at higher frequencies. The RMS (root-mean-square) of flow rate oscillations defined as 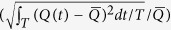, where 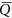 is the mean flow rate and *T* is the averaging time window, is shown in Fig. 4(b). The RMS fluctuation could be one order of magnitude higher in the stenosed vessels than in the non-stenosed vessel. Fourier spectra of 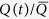 are plotted in Fig. 4(c) which shows an energy build-up at intermediate frequencies for the stenosed vessels. In contrast, energy decays continually for the non-stenosed vessels.

**Figure 5:**
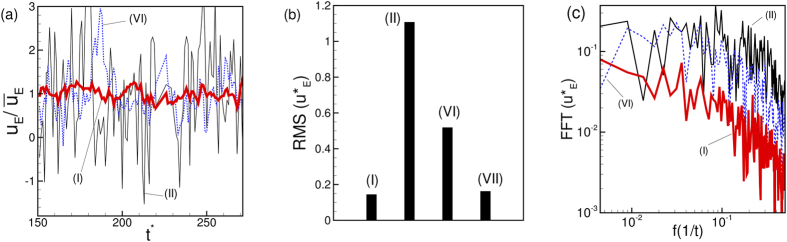
(**a**) Representative time-dependent Eulerian velocity 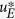 at a fixed radial distance 1.2 μm (near the edge of the CFL) from the wall but at three different streamwise locations: At a location far upstream (red thick line, location (I) as defined in Fig. 1), at the beginning of stenosis (black thin line, location II), and at the end of stenosis (blue dotted line, location VI). The Eulerian velocity has been scaled by the time-averaged velocity at the same location as 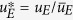. (**b**) RMS of fluctuations of 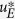 for the cases shown in (**a**). Also added is the RMS at a location far downstream marked as (VII). (**c**) Spectra of 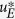 for the three cases shown in (**a**). Here *D* = 25 μm, *β* = 3, and *H*_*t*_ = 24%.

Also of interest is the instantaneous Eulerian velocity at certain locations. Figure 5(a) shows the streamwise component of the Eulerian velocity at a fixed radial distance of 1.2 μm from the wall of the vessel and at three different streamwise locations: far upstream (location I as defined in Fig. 1), at the beginning of the stenosis (location II), and end of stenosis (location VI). The velocity is scaled by the local time-averaged velocity 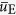 as 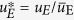. The RMS of fluctuations of 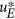 is shown in Fig. 5(b) for the above three locations, and also for a far downstream location. The fluctuations in the Eulerian velocity are significantly higher near the stenosis than those far away from the stenosis. Furthermore, close to the stenosis, the fluctuations at the upstream location II are higher than those at the downstream location VI. The Fourier spectra of 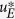 for the three locations are shown in Fig. 5(c). A continuous decay of 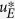 is observed far from the stenosis. In contrast, the spectra at locations II and VI show higher energy at intermediate to high frequency range. Between locations II and VI, the former shows higher energy than the latter at higher frequency range.

**Figure 6:**
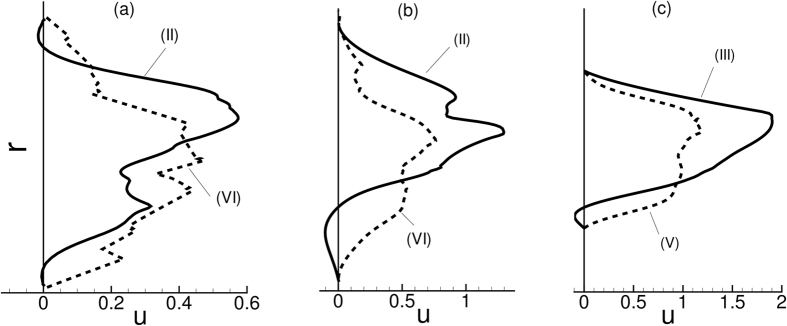
Upstream flow reversal in presence of RBCs: Shown here are instantaneous velocity profiles up- and downstream a symmetric stenosis (*D* = 25 μm, 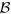 = 84%, *β* = 3, *H*_*t*_ = 24%). (**a**,**b**,**c**) refer to three different time instants. Continuous lines represent upstream velocity profiles at locations (II) or (III) as indicated (see Fig. 1 for locations), and dash lines represent downstream profiles at locations (V) or (VI). Here *r* is the radial location.

A closer inspection of the instantaneous velocity in Fig. 5(a) suggests an occasional reverse flow at the upstream location II. In contrast, the downstream flow remains unidirectional. The instantaneous velocity profiles are shown in Fig. 6 to further illustrate this point. In this figure, a reverse flow is evident in the upstream velocity profile, but not for the downstream profile. As shown in the figure, the reverse flow is highly transient and is observed at different azimuthal locations at different times. Importantly, no flow reversal would occur in the present geometry in absence of the cells. The reverse flow occurs when the cells squeeze through the stenosis displacing the near-wall fluid both up- and downstream. Additionally, cell jamming upstream the neck causes an instantaneous build-up of a high pressure and, hence, a local adverse pressure gradient leading to the observed flow reversal.

The results in Figs 5 and 6 suggest that an asymmetry in the flow quantities exists along the length of the stenosis. Such an asymmetry is further manifested in the averaged quantities as discussed below.

**Figure 7:**
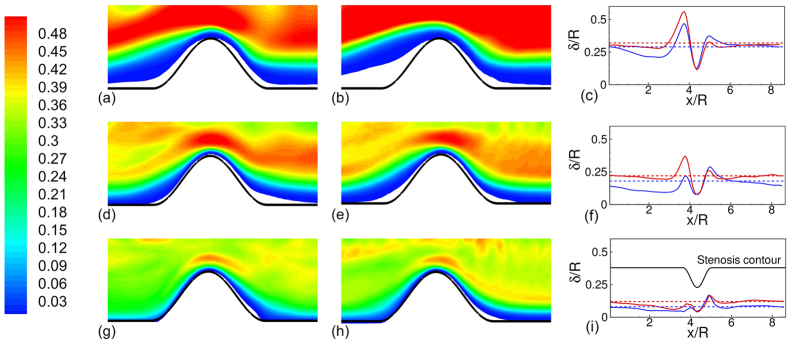
Time- and azimuthally-averaged RBC distribution at *β* = 1 (**a,d,g**) and 4 (**b,e,h**), and spatial variation of CFL thickness *δ/R* (**c,f,i**) for *D* = 11 μm (**a–c**), 17 μm (**d–f**), and 25 μm (**g–i**). For the RBC distribution, contours are plotted from 0 (blue) to 0.5 (red) with 0.01 increment. For the CFL thickness, dotted lines are for non-stenosed vessels, continuous lines for stenosed vessels, *β* = 1 (blue) and 4 (red). Here 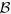 = 84%, and *H*_*t*_ = 24%. Here *R* is the vessel radius in non-stenosed section.

The mean RBC spatial distribution and CFL thickness are shown in Fig. 7. In general, the CFL thickness is larger immediate up- and downstream, but is significantly smaller at the throat. More importantly, the cell distribution and the CFL thickness are not identical up- and downstream the stenosis. For *D* = 11 μm, the upstream CFL is much wider than the downstream one. The width of the upstream CFL increases with increasing pressure gradient. As noted previously in Fig. 2, the RBCs flow in a single file in *D* = 11 μm tube in presence of the stenosis. Immediately before entering the stenosis, a cell squeezes creating a wider CFL. Cell deformation increases with increasing pressure gradient, which in turn widens the CFL even further. As the cell squeezes out of the constriction, it bounces back to regain the parachute shape, thereby causing a reduction in the CFL downstream. The asymmetry in the CFL variation is reversed in the *D* = 25 μm vessel where the upstream CFL is smaller than the downstream one. This is due to cell crowding upstream as noted earlier.

**Figure 8:**
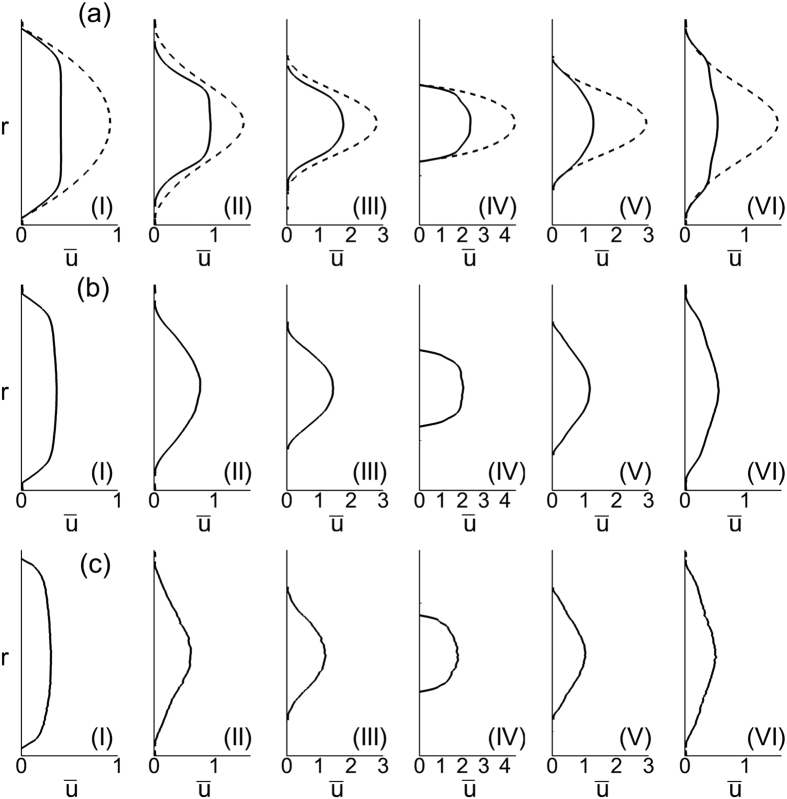
Average velocity profiles (*ū* vs. *r*) for symmetrically stenosed vessels (**a–c**) at different locations. *D* = 11 μm (**a**), 17 μm (**b**) and 25 μm (**c**). The dash line is the velocity profiles for pure plasma. (I) to (VI) correspond to different locations in the vessel as shown in Fig. 1. Here 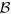 = 84%, *β* = 1, and *H*_*t*_ = 24%.

The average velocity profiles are shown in Fig. 8 at different streamwise locations. The well-known plug-flow profile is observed far up- and downstream (location I as marked in Fig. 1). However, differences exist between up- and downstream velocity profiles (e.g., at locations II and VI in the figure) near the stenosed region. At similar distances from the throat, the downstream profile is flatter than the upstream profile. The asymmetry arises due to the presence of the cells. For a Newtonian fluid the velocity profiles up- and downstream would be symmetrical in absence of inertia.

**Figure 9:**
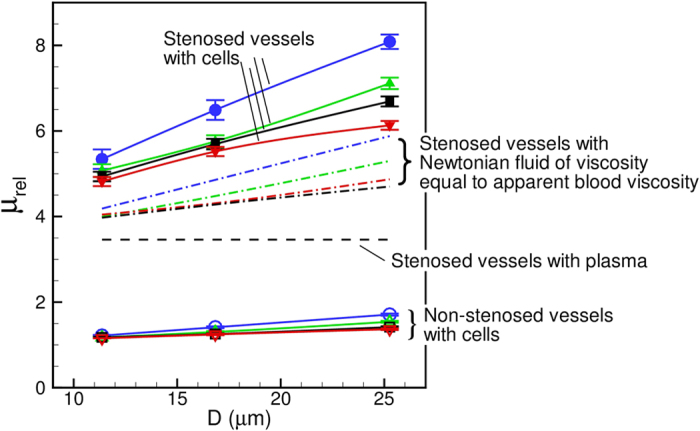
Apparent viscosity of blood showing a significant enhancement of the Fahraeus-Lindqvist effect in the stenosed vessels. Lines with unfilled symbols are for non-stenosed tubes with RBCs, and lines with filled symbols are for stenosed tubes (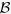 = 84%, *H*_*t*_  ≈ 22–24%) with RBCs, for various values of 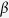 = 1 (O, blue), 2 (Δ, green), 3 (◻, black), 4 (∇, red). Dash line is for stenosed tubes with plasma only. Dash-dot lines are for stenosed tubes with Newtonian fluids having viscosities same as the apparent viscosity of blood in non-stenosed tubes of the same diameters.

The most striking result from this study is that the Fahraeus-Lindqvist effect is significantly enhanced in stenosed vessels. This is shown in Fig. 9 where *μ*_*rel*_ is plotted against *D*. Four cases are considered in the figure: the non-stenosed tubes with the RBCs, the 84% stenosed tubes with the RBCs, and the stenosed tubes with plasma, and with Newtonian fluids of the viscosity equal to the apparent viscosity of blood in a non-stenosed tube of the same diameter. A significant increase in *μ*_*rel*_ is observed in the stenosed vessels in presence of RBCs. For the nominal diameter of 25 μm, *μ*_*rel*_ in the 84% stenosed tube is more than four times that in the non-stenosed tube. The rate of increase of *μ*_*rel*_ with increasing *D*, as evident from the slope of the curves, is also much higher in the stenosed tubes compared to the non-stenosed tubes.

In order to see if the large increase in *μ*_*rel*_ in the stenosed tubes is due to geometric blockage only, we plot *μ*_*rel*_ that would occur when only plasma fluid is flown without any cell (dash line in the figure). While *μ*_*rel*_ is observed to increase due to the blockage effect, it is significantly below the values obtained in presence of the RBCs. Then, we consider the flow of Newtonian fluids of a viscosity equal to the apparent viscosity of the cellular blood obtained for the non-stenosed tubes (dash-dot lines in the figure). Even with this higher apparent viscosity, the values are significantly less. Hence, the significantly elevated apparent viscosity is not just due to the flow blockage. Apparently, it is due to the hydrodynamic interaction between the vascular geometry and the blood cells.

**Figure 10:**
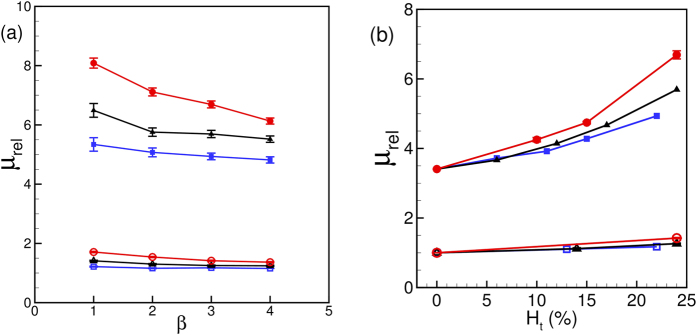
(**a**) Effect of mean pressure gradient *β* and (**b**) hematocrit *H*_*t*_ on the apparent viscosity for non-stenosed (continuous lines with unfilled symbols), and 84% stenosed tubes (continuous lines with filled symbols) for *D* = 11 (◻), 17 (Δ), and 25 μm (O). In (**a**) *H*_*t*_ = 24% is kept constant and *β* is varied; in (**b**) *β* = 3 is kept constant and *H*_*t*_ is varied.

Another important observation in Fig. 9 is the dependence of *μ*_*rel*_ on the driving pressure gradient *β*. Figure 10(a) shows *μ*_*rel*_ versus *β*. In general, *μ*_*rel*_ is observed to decrease with increasing pressure gradient, as a result of the shear-thinning nature of blood. The sensitivity of *μ*_*rel*_ to the changes in *β* increases with increasing diameter. However, the effect is more pronounced in the stenosed vessels. For example, for the non-stenosed vessels *μ*_*rel*_ is observed to increase by 6% in *D* = 11 μm tube and 25% in *D* = 25 μm as *β* is reduced from 4 to 1. In contrast, for the 84% stenosed tube, *μ*_*rel*_ increases by 11% for *D* = 11 μm and 31% for *D* = 25 μm tube. The increased influence of pressure gradient in the stenosed tubes also comes from the shear-thinning nature of blood: A reduced flow rate in the stenosed tubes causes a reduction in the mean shear rate, and hence an increase in *μ*_*rel*_. Additionally, Fig. 10(a) shows that the slope of the curves decreases with increasing pressure gradient at a faster rate in larger vessels and with stenosis. This is expected for a shear-thinning fluid and could be understood by considering the nature of RBC deformation. Since the volume and surface area of a cell remain constant, it can be deformed up to a certain limit. In the case of the smallest *D*, the cells are significantly deformed due to the geometric blockage even at the lower values of *β*. Increasing *β* for the smallest *D* does not significantly increase the amount of RBC deformation. Consequently, *μ*_*rel*_ reaches a saturation at higher *β* in the small vessels. For larger *D*, the intercellular space allows the suspension to compress more with increasing *β* and, hence, allows a greater change in *μ*_*rel*_. The saturation of *μ*_*rel*_ with increasing flow rate was also noted^40^ for RBCs flowing in capillary vessels.

Variation of *μ*_*rel*_ with hematocrit *Ht* is shown in Fig. 10(b). In general, *μ*_*rel*_ increases with increasing *H*_*t*_; however, the rate of increase is significantly higher for the stenosed vessels at larger diameter. As noted earlier in Fig. 2, the RBCs flow in a single-file manner in the smallest stenosed tube. Here *H*_*t*_ can be increased only up to a certain limit, and increasing *H*_*t*_ does not alter the single-file motion. For the larger vessels, increasing *H*_*t*_ causes a greater reduction in the CFL thickness. In addition, the RBCs crowd upstream the stenosis (Fig. 2) further reducing the CFL, and, hence, rapidly increasing the flow resistance.

The variation of the CFL and cell distribution discussed above helps explain the enhancement of the Fahraeus-Lindqvist effect. The driving pressure gradient is spent in deforming and moving the cells against the fluid drag. For the stenosed vessels, a part of the external energy is spent in additional deformation that the cells experience as they squeeze through the stenosis. The reduced intercellular distance as multiple cells simultaneously squeeze through the stenosis also causes additional frictional loss. The CFL on the other hand provides a near-wall layer of a low viscosity fluid and alleviates the loss. For the smaller vessels, the large increase in CFL thickness observed upstream the constriction (Fig. 7(c,f)) compensates for the decrease in the CFL at the throat. In contrast, such a compensating effect is not observed in the larger vessels, where, instead the upstream CFL is reduced due to cell crowding. As a result, the flow resistance increases at a greater rate as the vessel diameter increases.

**Figure 11:**
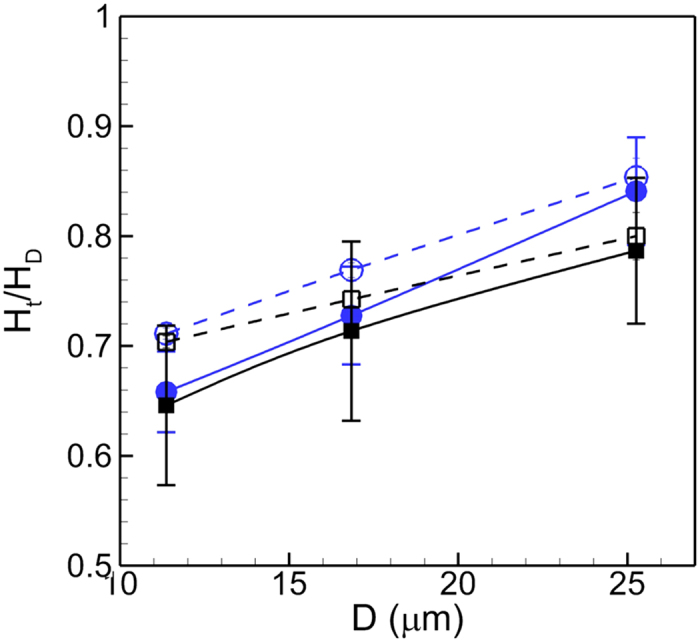
Comparison of the Fahraeus effect in 84% stenosed (filled symbols) and non-stenosed (unfilled symbols) vessels. Here hematocrit ratio *H*_*t*_/*H*_*D*_ is shown as a function of tube diameter *D* for *β* = 1 (O, blue), and 3 (◻, black), for *H*_*t*_ = 24%. Error bars represent absolute errors.

The Fahraeus effect is shown in Fig. 11 where the ratio of the tube hematocrit to discharge hematocrit *H*_*t*_*/H*_*d*_ is plotted against vessel diameter. The hematocrit ratio is computed as the ratio of the average blood velocity to the average RBC velocity. In general, *H*_*t*_*/H*_*d*_ is reduced in presence of a stenosis compared to its values in non-stenosed vessels. The difference in *H*_*t*_*/H*_*d*_ values in the stenosed and non-stenosed vessels is greater in smaller vessels due to the conversion of the multi-file motion to single-file motion as noted before in Fig. 2. The difference decreases with increasing vessel diameter, as the multi-file motion of the RBCs is recovered in the stenosed vessels, and CFL is reduced due to cell crowding. Also interesting to note is the effect of the mean pressure gradient *β* on *H*_*t*_*/H*_*d*_. For the smallest vessel, the effect of *β* is less as the cells are maximally deformed by the geometric blockage of the vascular wall. As the vessel diameter is increased, the multi-file motion is established and the intercellular space allows the suspension to be compressed more with increasing *β* leading to a reduction in the hematocrit ratio.

**Figure 12:**
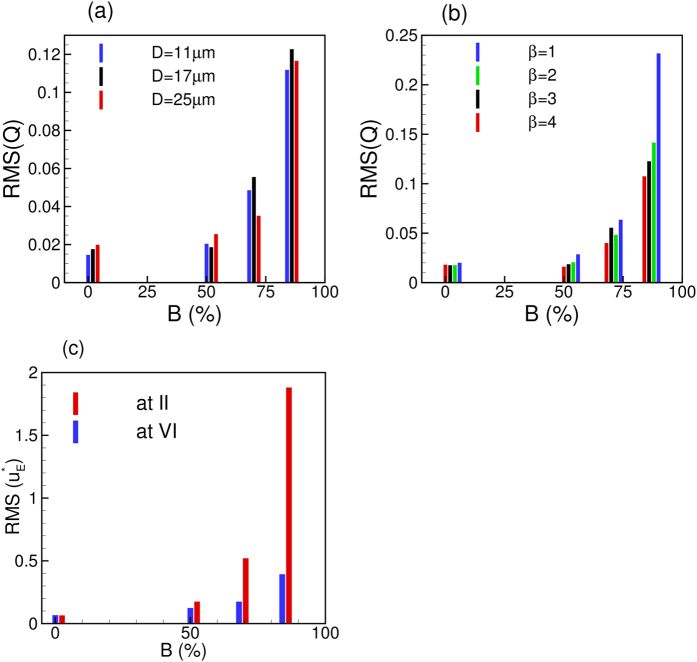
(**a**) Effect of area blockage 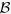 on RMS of flow rate oscillations for different vessels at constant *β* = 3. (**b**) Effect of 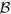 on RMS(*Q*) for different β but at constant *D* = 17 μm. (**c**) Effect of 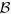 on Eulerian velocity fluctuations at locations II (upstream) and VI (downstream). Here *D* = 17 μm and *β* = 3. For all cases, *H*_*t*_ = 23%.

We now consider the effect of varying the area blockage 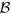 for symmetric constrictions. Fig. 12(a) shows the effect of 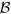 on the RMS of *Q*(*t*) for different vessels. The RMS remains nearly the same up to 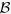 ≈ 50%, but increases rapidly thereafter. This is due to the conversion of the continuous flow of cells in to the discrete motion with increasing blockage. Figure 12(b) shows the effect of 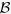 on flow oscillations for different values of *β*. Here also we observe that the effect of constriction is not felt up to 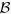 ≈ 50%, but the RMS rapidly increases thereafter. Similar observation can also be made for the Eulerian velocity fluctuations as shown in Fig. 12(c). This figure shows that the upstream velocity fluctuations increase at a faster rate than the downstream fluctuations. This, in turn, leads to an enhancement of the asymmetry in flow characteristics across the stenosis with increasing 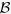. Furthermore, no flow reversal is observed for 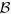 = 50%, but a transient flow reversal is observed upstream for 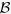 = 68 and 84%.

**Figure 13:**
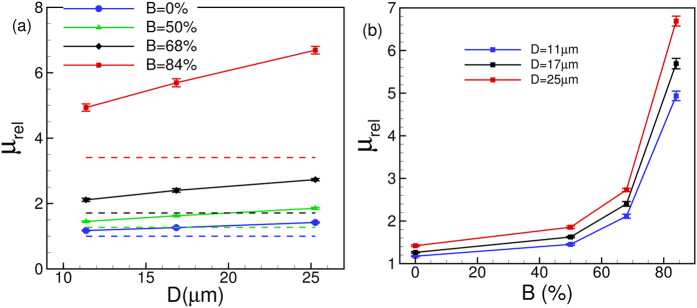
(**a**) Effect of 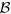 on the Fahraeus-Lindqvist effect. The dash lines are for plasma. (**b**) *μ*_*rel*_ versus 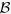 for different vessels.

Figure 13 shows the effect of 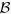 on *μ*_*rel*_. Both the values of *μ*_*rel*_ and slopes of the curves increase with increasing 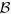 leading to an enhancement of the Fahraeus-Lindqvist effect. The enhancement is observed for all values of 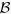, although the phenomenon is more pronounced for higher values. This is further illustrated in Fig. 13(b) where *μ*_*rel*_ versus 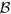 is plotted. For 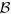 up to 50% only a modest increase in *μ*_*rel*_ is observed; thereafter *μ*_*rel*_ increases very rapidly.

An interesting question that is raised is whether the relative viscosity would remain unchanged if the physical opening at the throat area remains constant irrespective of the nominal size of the vessel. The answer is no, as evident from Fig. 13. For instance, a 64% stenosis in a 11 μm vessel and a 84% stenosis in a 17 μm vessel have the same area opening at the throat. However, *μ*_*rel*_ is nearly three times higher in the larger tube than in the smaller one. This observation further supports what is already explained earlier that the upstream cell crowding and the reduction in the CFL lead to a rapid increase in the apparent viscosity in larger vessels.

**Figure 14:**
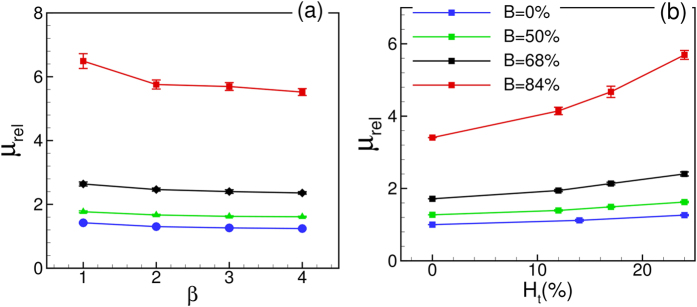
Effect of (**a**) *β* and (**b**) *H*_*t*_ on *μ*_*rel*_ for various 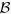. In (**a**) *H*_*t*_ = 23%, and in (**b**) *β* = 3. For all cases *D* = 17 μm.

The effect of *H*_*t*_ and *β* on *μ*_*rel*_ for different values of 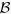 is shown in Fig. 14. For all cases, *μ*_*rel*_ is observed to increase with increasing *H*_*t*_ and decrease with increasing *β*. Shear-thinning effect gets more pronounced with increasing area blockage.

**Figure 15:**
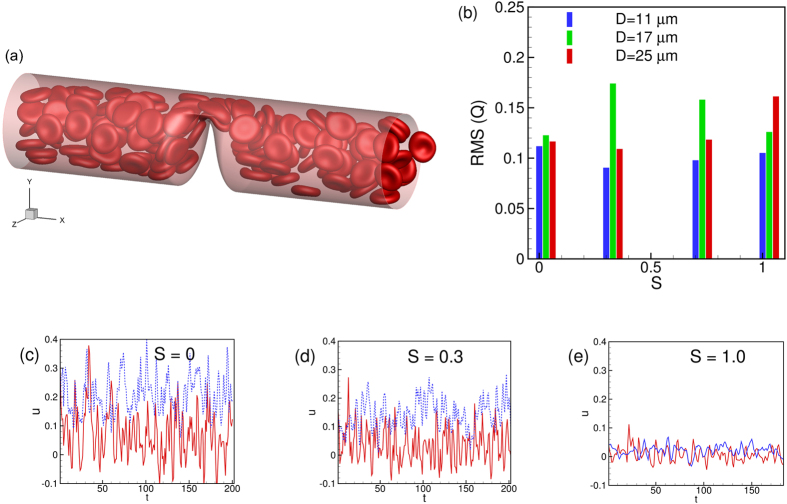
(**a**) A snapshot of cell motion through an asymmetric stenosis (*D* = 25 μm, 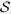 = 1, *β* = 1, *H*_*t*_ = 24%). (**b**) Effect of 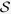 on RMS of flow rate. (**c**,**d**,**e**) shows the effect of 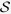 on time-dependent Eulerian velocity at locations II (continuous red line) and VI (dotted blue line).

We now consider varying the degree of asymmetry 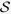 while keeping the area blockage 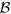 fixed at 84%. An instantaneous cell distribution for an asymmetric constriction at 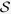 = 1 is shown in Fig. 15(a). Also presented is the RMS of *Q*(*t*) which does not show a significant change with respect to changing 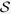. Based on the results it appears that the area blockage, not the degree of asymmetry, causes the enhanced flow oscillations. Figure 15(c–e) shows the time-dependent velocity at upstream location II and downstream location VI for three different values of 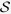 = 0, 0.3 and 1. The difference between the upstream and downstream velocities decreases with increasing 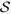. For 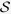 = 0 and 0.3, flow reversal occurs only upstream. But for 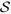 = 0.7 and 1, flow reversal occurs both up- and downstream. Hence the asymmetry in flow characteristics along the stenosis length decreases with increasing 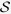.

**Figure 16:**
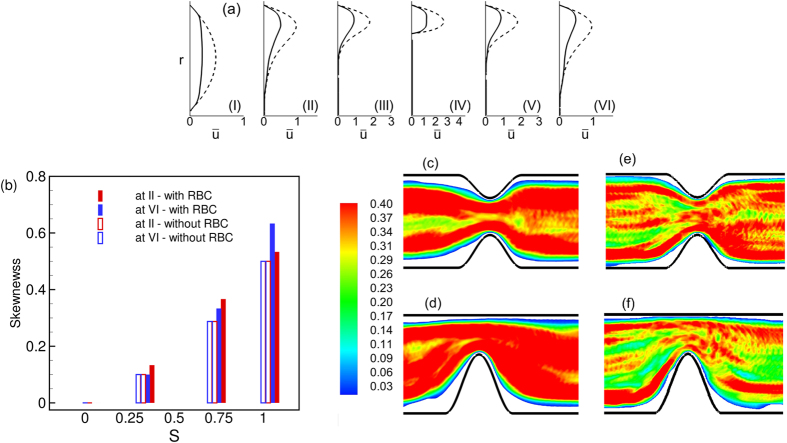
(**a**) Average velocity profiles (*ū* vs. *r*) at different streamwise locations for 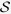 = 1, *D* = 17 μm. (**b**) Skewness of velocity profiles as a function of 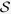 at locations II and VI. (**c**,**d**) RBC distribution in *D* = 17 μm for 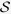 = 0.3 and 1, respectively. (**e,f**) Same but in *D* = 25 μm.

Figure 16(a) shows the mean velocity profiles along the streamwise locations. The velocity profile is now highly skewed, but the difference between up- and down-stream profiles is reduced. The skewness is plotted in Fig. 16(b) as a function of 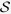 at up- and downstream locations. While the skewness increases significantly with increasing 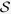, there is no significant difference between the up- and downstream values. The effect of 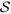 on the mean RBC distribution is shown in Fig. 16(c–f). The asymmetry in the variation of the CFL thickness along the stenosis length is observed to decrease with increasing 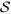. Also, for 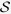 = 1 the CFL is observed to decrease near the vascular wall opposite to the stenosis.

**Figure 17:**
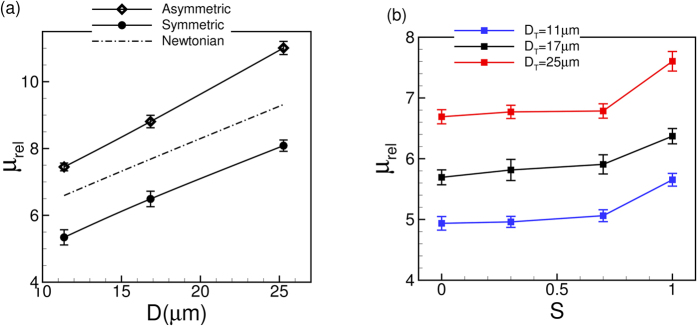
Comparison of the Fahraeus-Lindqvist effect in symmetric and asymmetric stenosis. (**a**) Variation of *μ*_*rel*_ with respect to *D* for 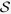 = 0 and 1. The dash-dot line is the relative viscosity obtained in the asymmetric stenosis for a Newtonian fluid having viscosity equal to the apparent blood viscosity in non-stenosed tubes. (**b**) Variation of *μ*_*rel*_ with respect to S for different vessels.

The relative viscosity variations with increasing tube diameter for symmetric and asymmetric stenosis are compared in Fig. 17(a). As much as 40% increase in the relative viscosity is observed at 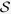 = 1 compared to the symmetric stenosis. Also shown in the figure is relative viscosity obtained in the asymmetric stenosis for a Newtonian fluid having a viscosity equal to the apparent blood viscosity in the non-stenosed tube. As noted earlier, the Newtonian fluid significantly underpredicts the apparent viscosity obtained in presence of the RBCs. Furthermore, the slope of the curve in the asymmetric stenosis in presence of the RBCs is higher than that obtained for the symmetric stenosis. This result implies an additional augmentation of the Fahraeus-Lindqvist effect in asymmetric cases. One factor that causes such augmentation is the reduction of the CFL in the wall opposite to the stenosis as noted above. Unlike *μ*_*rel*_, the hematocrit ratio *H*_*t*_*/H*_*d*_ increases by a small amount (≤2.5%) as S increases from 0 to 1; hence, these data are not presented.

## Conclusion

**Figure 18:**
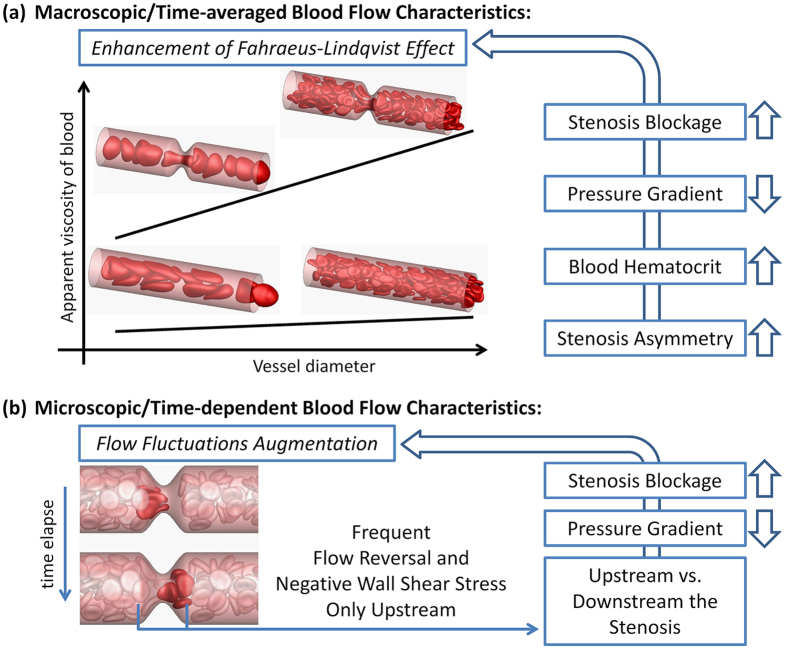
Summary of the findings.

A numerical study of the flow of deformable red blood cells in stenosed microvessels is presented. We consider the effect of varying the vessel diameter, area blockage, degree of asymmetry, pressure gradient, and hematocrit on the apparent blood viscosity, and mean and instantaneous flow characteristics. A summary of the findings of this study is presented schematically in Fig. 18.

The qualitative nature of the cellular motion is altered in the presence of a stenosis. For the smallest diameter, the multifile motion is converted to a single file motion. For larger diameters, the continuous flow of the cells is converted in to a discrete flow. For the latter case, a crowding of the cells upstream the stenosis is observed. While we consider the geometry rather small so as to allow for a large number of simulations within a reasonable computational cost, several novel findings are obtained in this study.

The discrete motion of the cells through a stenosis causes large time-dependent fluctuations in flow properties. In particular, the RMS of flow rate fluctuations in presence of a stenosis could be an order of magnitude higher than that in non-stenosed vessels. The flow rate drops when cells are about to enter the stenosis, and increases when they come out of the stenosis. Several folds increase in Eulerian velocity fluctuation is also observed in the stenosed vessel compared to the non-stenosed case. Furthermore, the velocity fluctuation immediate upstream is much higher than that downstream. Surprisingly, a transient flow reversal is observed upstream a stenosis but not downstream.

Physiological implications of the flow oscillations and upstream reversal could be significant. Such transient flow reversals make the instantaneous wall shear-stress (WSS) also highly fluctuating and, often negative. The fluctuating and negative WSS is known to play a significant physiological role in terms of endothelial cell response. Additionally, the flow reversal also causes an increase of the residence time of flowing platelets, leukocytes and macromolecules in the upstream section of the stenosis. The results could also be useful to improve our understanding of leukocyte extravasation which is sensitive to local flow characteristics.

The present results underscore the differences between micro- and macro-vascular hemodynamics. In macrovascular stenosis, blood behaves as a Newtonian fluid and the flow is inertia dominated. Under these conditions, a flow reversal appears downstream^1^,^2^,^3^,^4^,^5^. Furthermore, the flow and WSS oscillations arise due to the pulsatile and a weakly turbulent nature of the blood flow^2^,^3^,^4^,^5^. In contrast, in microvascular stenosis, the oscillations and upstream flow reversal arise entirely due to the presence of the cells.

The asymmetric distribution of the cells also results in an asymmetric variation of the cell-free layer along the length of the stenosis. The CFL is reduced significantly at the throat of a stenosis in all vessels, but is increased away from the throat. For the smaller vessels, the upstream CFL is wider than the downstream one. In contrast, the situation is reversed in the larger vessels. The asymmetry in the cell distribution also results in an asymmetry in the average velocity. The downstream velocity profiles are flatter than the upstream ones. Consequently, the average WSS upstream is expected to be reduced compared to that downstream.

Another important finding of the study is that the Fahraeus-Lindqvist effect is significantly enhanced in the stenosed vessels. The apparent viscosity *μ*_*rel*_ is observed to increase by several folds compared to that in non-stenosed vessels. Also, the slope of the *μ*_*rel*_-*D* curves is higher in the stenosed vessels. The large increase in *μ*_*rel*_ in stenosed vessels cannot be predicted by using the apparent viscosity of blood in non-stenosed vessels of same nominal diameters. The enhanced viscosity is explained using the observed flow characteristics that results from the interaction between the cells and the vascular geometry. The stenosed geometry also reduces the tube to discharge hematocrit ratio, thereby affecting the Fahraeus effect. These findings are also physiologically important: A significantly elevated apparent viscosity and a reduced hematocrit imply a reduced tissue perfusion and oxygenation. Such a condition is referred to as ischemia and could lead to organ failure^6^,^7^,^8^,^9^,^10^,^11^,^12^.

Our results also suggest that the area blockage affects the rheology and hemodynamics more severely than the geometric asymmetry. A moderate increase in flow asymmetry and apparent viscosity is observed up to 50% blockage. Above 50% blockage, the flow asymmetry and apparent viscosity are observed to rapidly increase due to more discrete nature of the cellular flow. The RMS of flow rate oscillations does not change significantly with a change in the degree of asymmetry. Also, the asymmetry in the flow characteristics appears to be reduced as the geometric asymmetry is increased. The apparent viscosity increases by a modest amount for 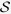 up to ~0.7, but more rapidly thereafter.

## Additional Information

**How to cite this article**: Vahidkhah, K. *et al*. Flow of Red Blood Cells in Stenosed Microvessels. *Sci. Rep.* **6**, 28194; doi: 10.1038/srep28194 (2016).
